# Predominant Non-Traditional or “Exotic” Pet Species and Affected Body Systems and Sense Organs at a Veterinary Referral Center: An Eleven-Year Retrospective Study

**DOI:** 10.3390/ani16193146

**Published:** 2026-10-08

**Authors:** Daniel Simões da Silva, Sheila Canevese Rahal, Heloisa Coppini de Lima, Gabriel Corrêa de Camargo, Ricardo Shoiti Ichikawa, Amanda de Barros Piffer, Alessandra Melchert, Renato Moran Ramos

**Affiliations:** 1 School of Veterinary Medicine and Animal Science (FMVZ), São Paulo State University (UNESP), Prof. Doutor Walter Mauricio Correa s/n, Botucatu 18618-681, SP, Brazil; daniel.simoes@unesp.br (D.S.d.S.); sheila.canevese-rahal@unesp.br (S.C.R.); heloisa.coppini@unesp.br (H.C.d.L.); gabriel.c.camargo@unesp.br (G.C.d.C.); ricardo.ichikawa@unesp.br (R.S.I.); amanda.piffer@unesp.br (A.d.B.P.); alessandra.melchert@unesp.br (A.M.); 2 Companion Animal Clinic, College of Veterinary Medicine, Midwestern University, Glendale, AZ 85308, USA

**Keywords:** exotic pets, birds, mammals, reptiles, epidemiology

## Abstract

This study characterized the non-traditional or “exotic” pets most frequently admitted to a referral center (Center for Medicine and Research in Wild Animals–CEMPAS) located in Botucatu, São Paulo state, Brazil, and identified the main reasons for admission according to affected body systems or sense organs. A retrospective review of medical records was performed over 11 years, and data were grouped into four vertebrate classes: reptiles, mammals, amphibians, and birds. From 2014 to 2019, bird admissions exceeded mammals (*p* = 0.0001), whereas no significant difference was observed from 2020 to 2024 (*p* = 0.625). Reptiles were the least represented, and no amphibians were recorded in any year. In the bird class (*n* = 961), cockatiels (*Nymphicus hollandicus*) were the most common species (47.76%), followed by domestic chickens (*Gallus gallus domesticus*) (15.19%), and blue-fronted Amazons (*Amazona aestiva*) (8.01%). The digestive, integumentary, and musculoskeletal systems were the most affected. The mammal class accounted for 882 admissions, with rabbits (*Oryctolagus cuniculus*) predominating (52.38%), followed by guinea pigs (*Cavia porcellus*) (17.57%), brown rats (*Rattus norvegicus*) (10.77%), and Syrian hamsters (*Mesocricetus auratus*) (6.00%). In these animals, digestive disorders were most common, followed by integumentary, musculoskeletal, and reproductive conditions. The reptile class accounted for 164 admissions, mainly tortoises (70.12%) and turtles (18.90%), with the integumentary, musculoskeletal, and digestive systems most frequently affected. Overall, birds and mammals were the most commonly admitted non-traditional or “exotic” pets, while the digestive and integumentary systems were the most frequently affected across species, emphasizing the value of regional epidemiological data for improving exotic animal veterinary care.

## Simple Summary

Exotic pets have become increasingly popular, creating a growing demand for appropriate veterinary care. This study reviewed 11 years of medical records from the Center for Medicine and Research in Wild Animals (CEMPAS; Brazil) to identify the most common non-traditional or “exotic” pets presented and the main affected body systems and sense organs affecting them. Birds accounted for a substantial proportion of the admissions, with cockatiels being the most frequently represented, followed by chickens and parrots. Among mammals, rabbits were the most commonly presented species, followed by guinea pigs, rats, and hamsters. Reptiles, mainly tortoises and turtles, were less frequently presented. The most common health problems varied among classes, but digestive and integumentary disorders were the leading causes of veterinary visits overall. Unlike dogs and cats, many exotic animals hide signs of illness until their condition becomes severe, making appropriate veterinary attention essential. Studies like this may help improve prevention, diagnosis, and treatment by identifying the most prevalent affected body systems, thereby contributing to the welfare and quality of life of non-traditional or “exotic” pets.

## 1. Introduction

The term “exotic” pet can be difficult to define; in general, it refers to species that are considered unusual, uncommon, or nontraditional compared with conventional domestic animals, encompassing not only non-native species but also animals that may be unfamiliar or uncommon within a particular society [1]. Alternatively, the British Small Animal Veterinary Association prefers the term “non-traditional companion animal” to “exotic” pet for species not traditionally domesticated or kept as pets and whose welfare needs may be challenging to meet in domestic settings [2]. To ensure a more objective and balanced approach to identifying animals considered “exotic” pets and supporting decision-making between sellers and buyers, the EMODE system was developed to classify animals as “easy,” “moderate,” “difficult,” or “extreme” based on the challenges associated with their maintenance, particularly regarding animal welfare and human health and safety [3]. Similarly, the Responsible Pet Guide for Exotic Pet Ownership, developed by WWF (World Wildlife Foundation)-US and WWF-Japan, provides information on species suitability and the potential risks of “exotic” pet ownership, with particular consideration of animal welfare [4].

The ownership of “exotic” pets has increased worldwide, requiring appropriate veterinary care because the unique anatomical and physiological characteristics of each species [5,6,7]. The prevalence of “exotic” pets can vary significantly among countries and even among regions within the same country [6,8,9,10], highlighting the need for specific knowledge of the species most commonly kept in each location. Estimates of “exotic” pet ownership between 2010 and 2012 indicated rates of 34.2% in Europe, 63.2% in the United Kingdom, 44.0% in Germany, and 36.5% in the United States [11]. Along with the increasing availability of “exotic” pets, concerns regarding their trade have also grown, as demonstrated by a global analysis of the exotic pet trade based on scientific and gray literature and CITES trade data from the United Nations Environment Programme–World Conservation Monitoring Centre (UNEP-WCMC) for 2006–2012, which found that birds were the most frequently reported class in trade, followed by reptiles and, lastly, mammals [12].

Keeping “exotic” pets may be a way for people to express and practice forms of care that differ from those directed toward humans; however, this attitude may not align with conservation goals [13]. Owners often acquire “exotic” animals impulsively, with insufficient consideration given to husbandry requirements, veterinary care expenses, and potential welfare issues [5]. Additionally, new media have influenced pet acquisition and the pet trade [12]. A study using online questionnaires administered to 719 Brazilian reptile pet keepers found that the primary motivations for keeping reptiles—particularly snakes, lizards, and turtles—included emotional reasons, followed by entertainment and convenience [14].

Many “exotic” pet species are not domesticated and may exhibit wild or unpredictable behaviors, while their specialized behavioral, environmental, and husbandry requirements can be challenging for owners to adequately meet [15]. Among the differences in veterinary care between traditional and “exotic” pets, the need for hospitalization of the latter is often highlighted due to several factors: the severity of the condition, as many exotic species tend to mask clinical signs until they become critically ill; owner noncompliance with medication administration; and suboptimal diet and husbandry, which may contribute to the underlying cause of the illness [16].

Therefore, this study aimed to characterize non-traditional or “exotic” pets most frequently admitted to a referral center in São Paulo State, Brazil, and to identify the reasons for their admission based on the affected body systems, thereby generating information that may indirectly contribute to conservation efforts. The hypothesis was that admission frequencies would differ among non-traditional or “exotic” pet classes, with birds being the most commonly admitted in all years.

## 2. Materials and Methods

### 2.1. Animals

Medical records of non-traditional or “exotic” pets admitted to the Center for Medicine and Research in Wild Animals (CEMPAS) (22°53′17.6″ S, 48°29′56.6″ W), School of Veterinary Medicine and Animal Science, São Paulo State University (UNESP), Botucatu campus, São Paulo, Brazil, were retrospectively analyzed over an 11-year period (2014–2024). All animals had been evaluated by licensed veterinarians, who established the diagnoses based on physical examination and, when necessary, complementary diagnostic tests, including laboratory analyses and imaging examinations, as appropriate for each case.

All privately owned non-traditional or “exotic” pets included in this study were non-domesticated or non-traditionally domesticated species that are not conventionally kept as companion animals. Data were categorized into four vertebrate classes: reptiles, mammals, amphibians, and birds. For each class, the recorded variables included animal species; reason for admission based on health status or affected anatomical system (cardiovascular, digestive, endocrine, integumentary, lympho-hematopoietic, musculoskeletal, nervous, reproductive, respiratory, or urinary) or sense organ (hearing or vision); outcome; and year of occurrence. Animals with no clinically relevant disease or abnormality after clinical examination and appropriate diagnostic procedures were classified as clinically healthy. Sex and age were not evaluated because this information was unavailable for many animals, and several species included in the study do not exhibit readily discernible external sexual dimorphism.

In cases of recurrence of the same condition, data collection was limited to the outcome only; recurrent events were not recorded. If more than one body system was affected in an animal, all affected systems were recorded. Medical discharge was considered released from the hospital or medical care after treatment.

### 2.2. Statistical Analyses

Animal classes with zero frequency throughout the study period were excluded from all statistical analyses. The chi-square test of homogeneity was used to compare the percentage frequencies of animal classes admitted to the referral center across the study years, in order to assess whether the composition of admissions remained constant over time or showed significant temporal changes. Ninety-five percent confidence intervals were estimated for the annual proportion of each class to quantify the precision of these proportions. The intervals reflect variability in the admission profile rather than error or uncertainty in the recorded counts. To compare the number of admissions among animal classes within the two periods (2014–2019 and 2020–2024), data normality was assessed using the Shapiro–Wilk test, followed by one-way analysis of variance (ANOVA).

Additionally, the temporal trend in admitted animals by affected body system or sense organ was evaluated using simple linear regression, with year as the independent variable and the annual number of admitted animals as the dependent variable. This analysis was performed separately for each vertebrate class and for each of the 12 affected body systems/sense organs, as well as for healthy animals, over the 2014–2024 period. The same procedure was applied to evaluate temporal trends in the four outcomes (medical discharge, euthanasia, death, and loss to follow-up) within each vertebrate class. In both analyses, the slope coefficient, coefficient of determination (R^2^), and *p*-value were estimated for each regression, with trends considered statistically significant at *p* < 0.05. The temporal trend of the total number of admitted animals per class was evaluated using non-linear polynomial regression, comparing first-degree (linear), second-degree (quadratic), and third-degree (cubic) fits, with the best-fitting model selected based on the coefficient of determination (R^2^).

Statistical analyses were conducted using BioEstat 5.0, GraphPad Prism versions 4 and 8.4.2 (GraphPad Software, San Diego, CA, USA), and R version 4.1.0 (R Core Team). A significance level of 5% (*p* < 0.05) was adopted.

## 3. Results

**Figure 1:**
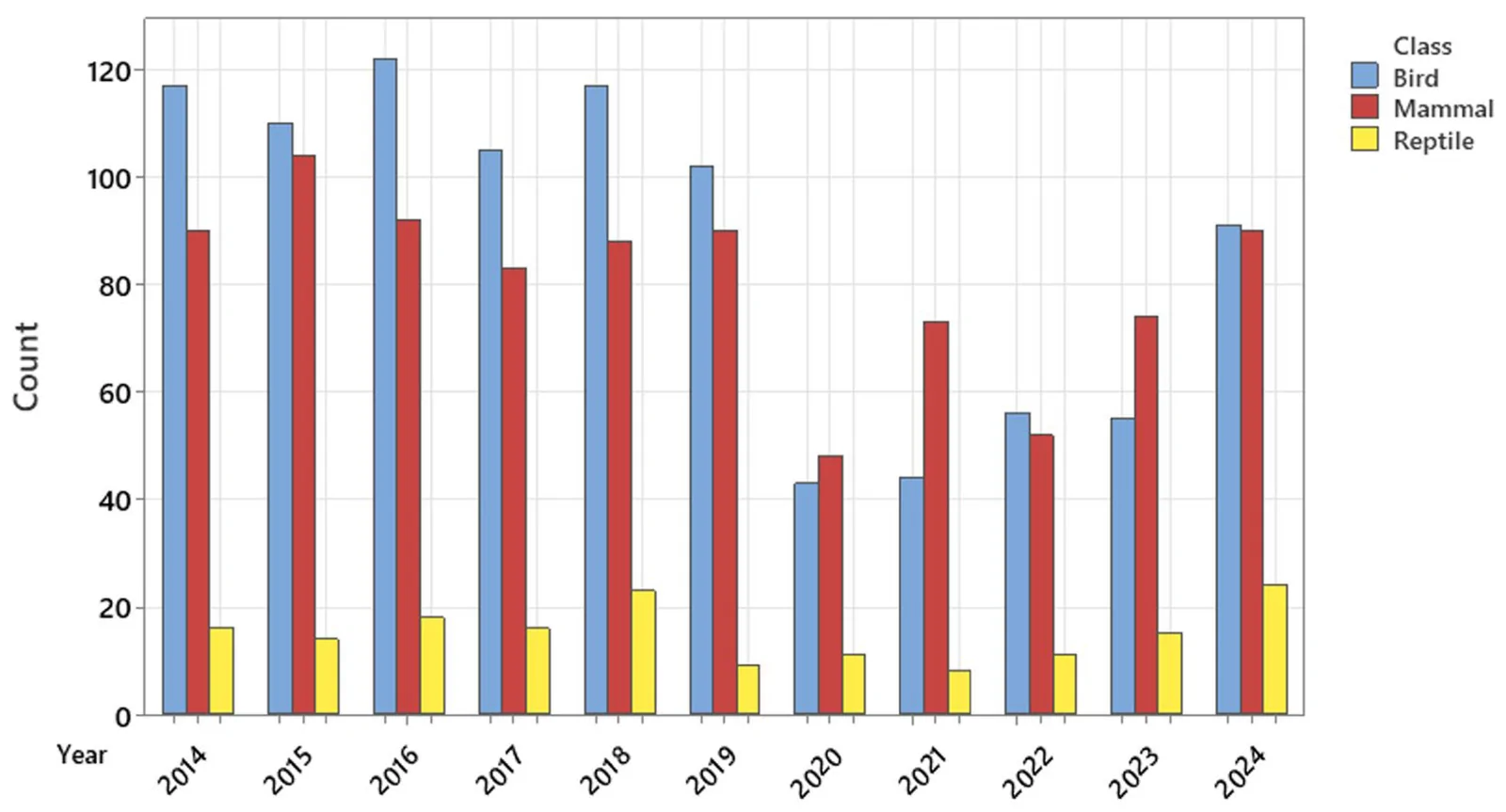
Annual number of birds, mammals, and reptiles admitted to the referral center from 2014 to 2024.

A total of 2007 individual animals were recorded, comprising 961 birds (47.9%), 882 mammals (44.0%), and 164 reptiles (8.2%), while no amphibians were recorded in any year. The proportional distribution of these classes varied significantly over time (χ^2^(20) = 31.78, *p* = 0.045). Birds predominated from 2014 to 2019, accounting for approximately half of admissions each year, while mammals ranked second and reptiles consistently represented the smallest proportion (Figure 1, Table 1). In 2020, 2021, and 2023, mammals accounted for the highest number of admissions, followed by birds, whereas reptiles remained the least frequently admitted class (Figure 1, Table 1). From 2014 to 2019, bird admissions were significantly higher than mammal (*p* = 0.0001) and reptile admissions (*p* < 0.0001), while mammal admissions exceeded reptile admissions (*p* < 0.0001). From 2020 to 2024, bird and mammal admissions did not differ (*p* = 0.625), whereas reptile admissions were lower than those of mammals (*p* = 0.0004) and birds (*p* < 0.002).

**Figure 2:**
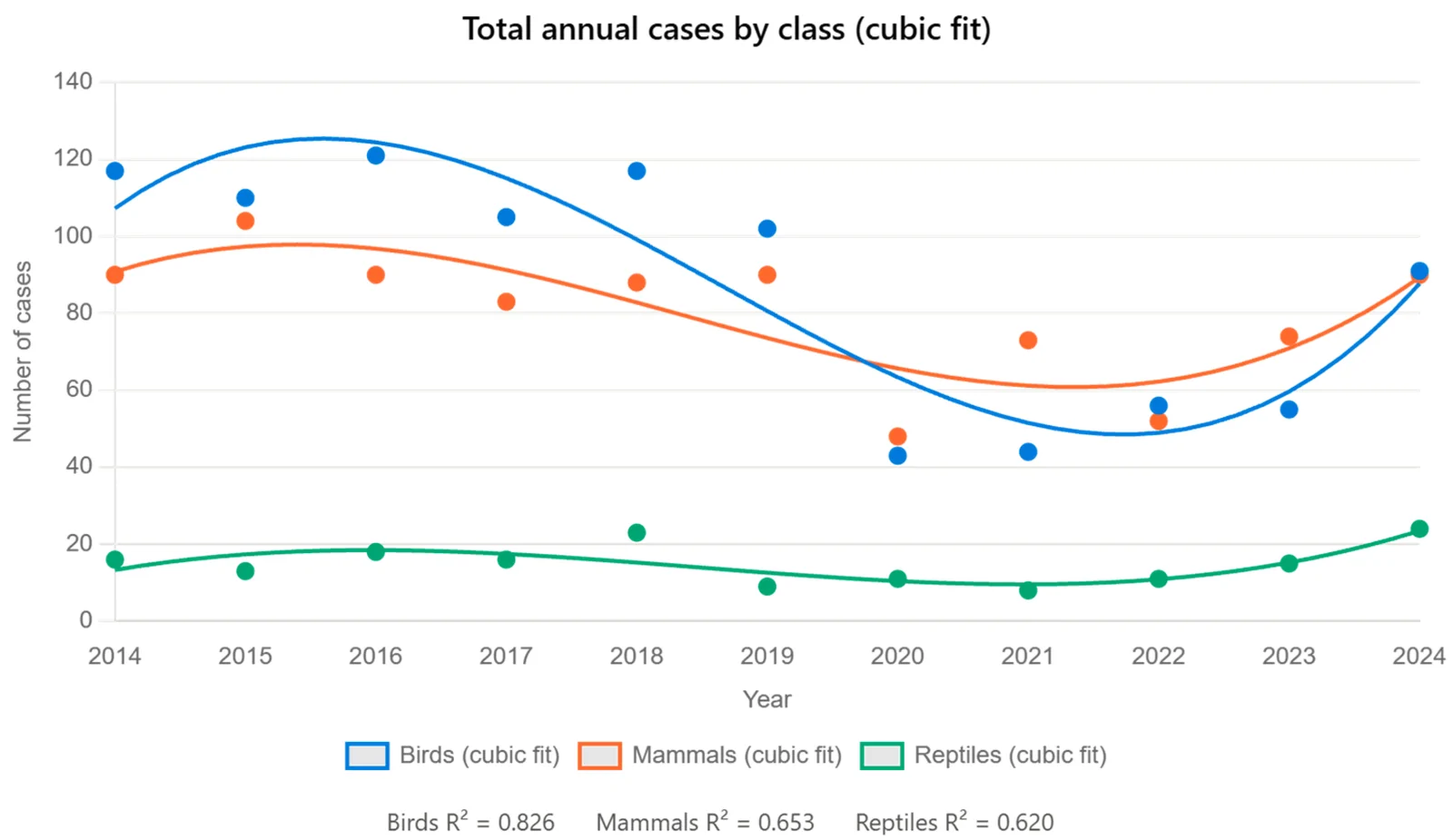
Temporal trends in animal admissions by vertebrate classes (birds, mammals, reptiles) over an 11-year period.

Polynomial analysis of the total number of admitted animals revealed a similar pattern across the three classes over the 11-year study period (Figure 2). Numbers of admitted animals remained relatively stable from 2014 to 2019, followed by a sharp decline in 2020 and a partial recovery in subsequent years, without returning to pre-2020 levels.

Among birds, admissions mainly consisted of cockatiels (*Nymphicus hollandicus*; 459/961), followed by domestic chickens (*Gallus gallus domesticus*; 146), blue-fronted amazons (*Amazona aestiva*; 77), and white-eyed parakeets (*Psittacara leucophthalmus*; 34). Bird admissions declined in 2020–2021, with partial recovery afterward. Among mammals, European rabbits (*Oryctolagus cuniculus*; 462/882), guinea pigs (*Cavia porcellus*; 155), brown rats (*Rattus norvegicus*; 95), and Syrian hamsters (*Mesocricetus auratus*; 53) were the most frequent. Reptile admissions largely consisted of chelonians, especially red-footed tortoises (*Chelonoidis carbonaria*; 113/164), followed by black-bellied slider *(Trachemys dorbigni*; 25), while other reptile species were admitted only occasionally. Table 2, Table 3 and Table 4 list the animal species in the following order: birds, mammals, and reptiles, respectively.

Most admitted animals presented at least one clinical abnormality, whereas clinically healthy individuals were uncommon (32 birds, 20 mammals, and 11 reptiles). Because some animals had more than one affected body system/sense organ, the total number of recorded conditions exceeded the number of individuals.

**Figure 3:**
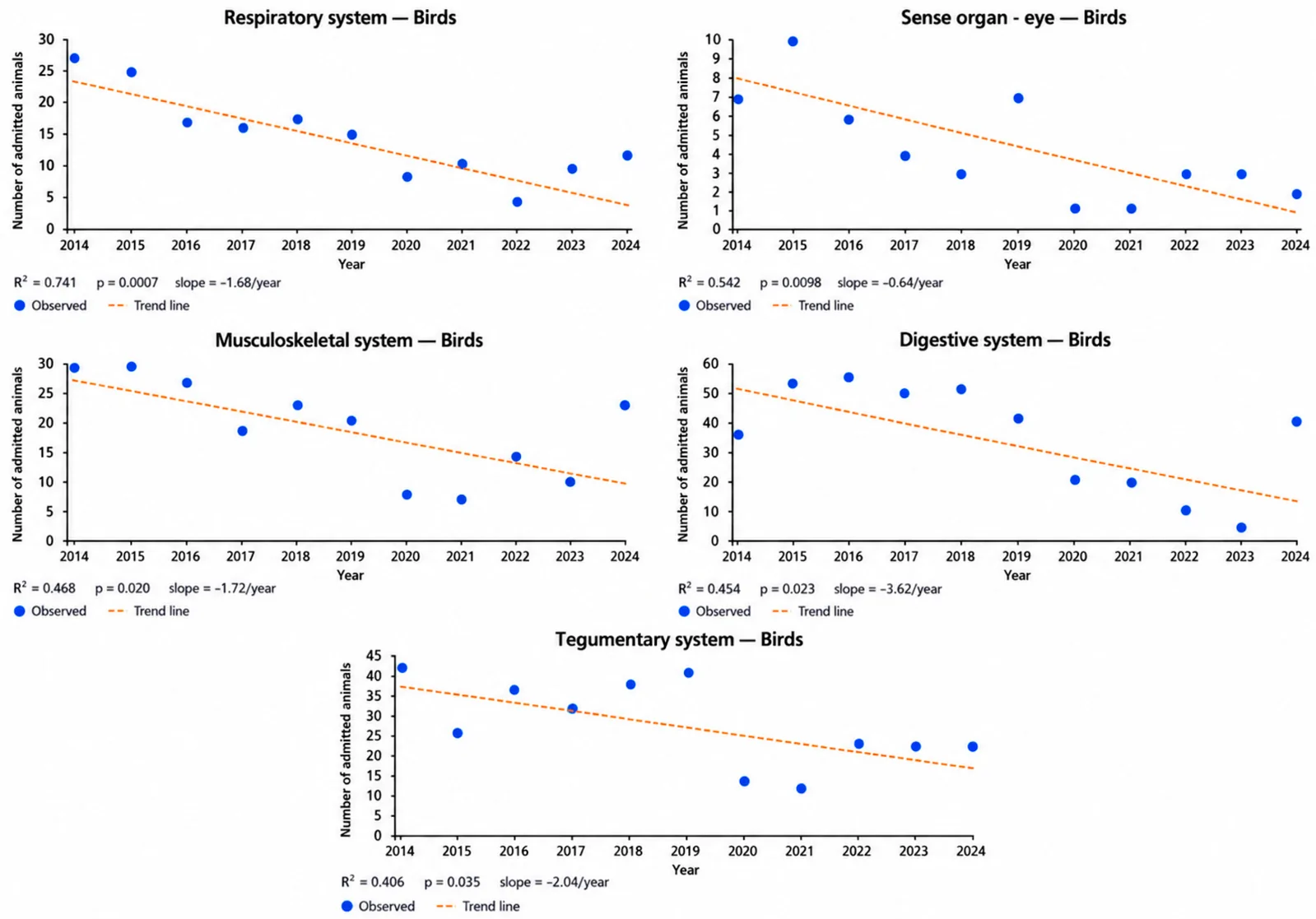
Significant declining trends in selected body systems/sense organs over an 11-year period in non-traditional or “exotic” pet birds.

In birds, the digestive (*n* = 383), integumentary (*n* = 310), musculoskeletal (*n* = 213), and respiratory (*n* = 163) systems were most frequently affected. Of the 12 body systems/sense organs evaluated, including healthy admissions, five showed significant declining trends over time: respiratory (R^2^ = 0.741, *p* = 0.0007), eye (R^2^ = 0.542, *p* = 0.0098), musculoskeletal (R^2^ = 0.468, *p* = 0.020), digestive (R^2^ = 0.454, *p* = 0.023), and integumentary (R^2^ = 0.406, *p* = 0.035) (Figure 3). No significant temporal trends were observed for the remaining categories (*p* > 0.05). The endocrine system was not recorded and was therefore excluded from regression analyses.

In mammals, digestive (*n* = 303) and integumentary (*n* = 288) disorders were the most common, followed by musculoskeletal (*n* = 126) and reproductive (*n* = 105) conditions. Only integumentary disorders showed a significant decline over time (R^2^ = 0.437, *p* = 0.027). Healthy condition, digestive, and nervous system records showed non-significant trends (*p* = 0.088, 0.068, and 0.069, respectively), whereas no significant temporal variation was observed for the remaining systems and sense organs (*p* > 0.05). Endocrine and lymphohematopoietic systems were excluded because of insufficient records for regression analysis.

In reptiles, integumentary (*n* = 57), musculoskeletal (*n* = 41), digestive (*n* = 40), and reproductive (*n* = 36) disorders predominated. Only integumentary disorders showed a significant decline over time (R^2^ = 0.395, *p* = 0.038). No significant trends were observed for healthy animals or the other body systems/sense organs (*p* > 0.05). Lympho-hematopoietic and urinary systems were not recorded and were therefore excluded from regression analyses.

**Figure 4:**
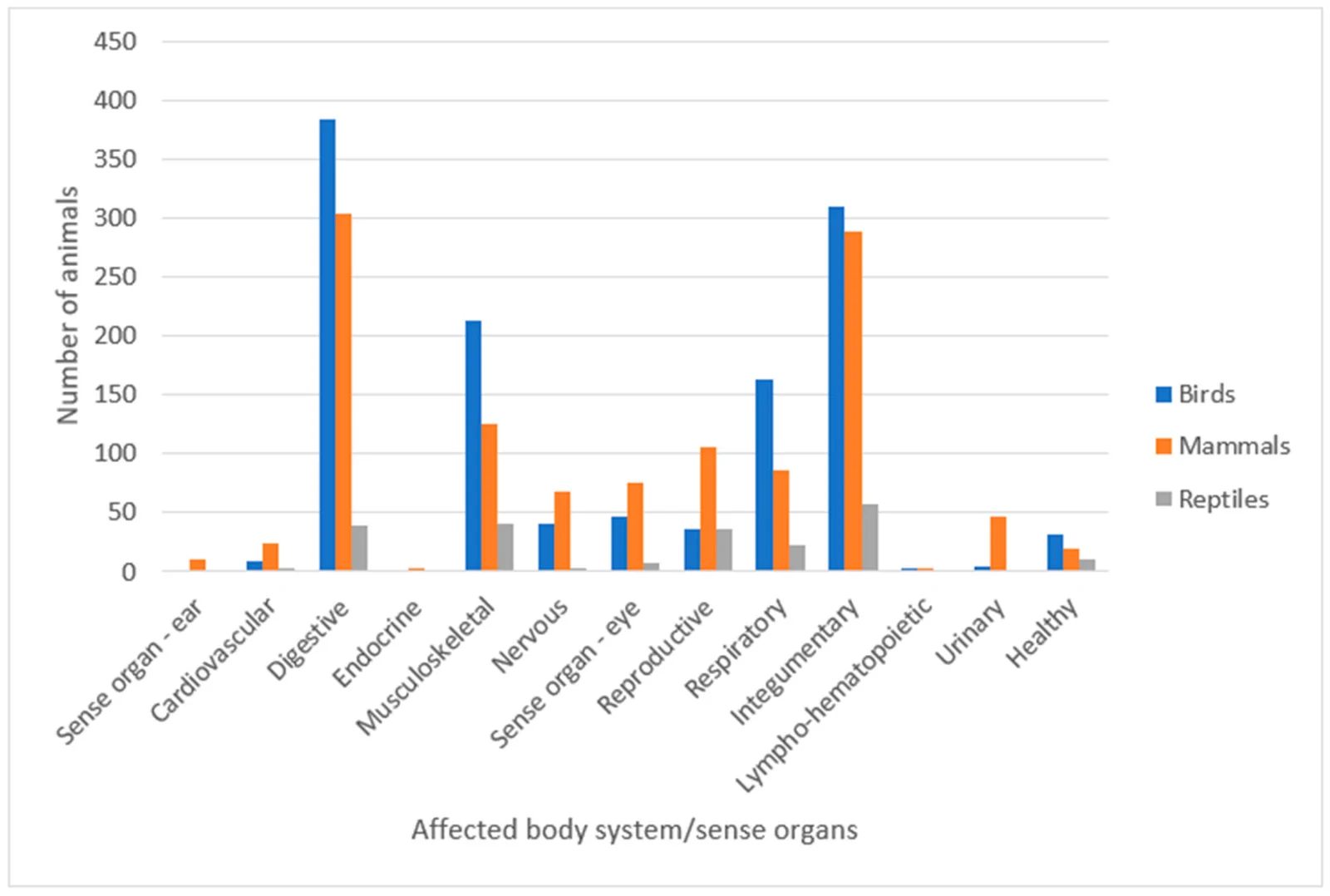
Health status or affected body system/sense organs in birds, mammals, and reptiles over an 11-year period.

Figure 4 shows the affected body systems/sense organs and health statuses of birds, mammals, and reptiles across all years.

**Figure 5:**
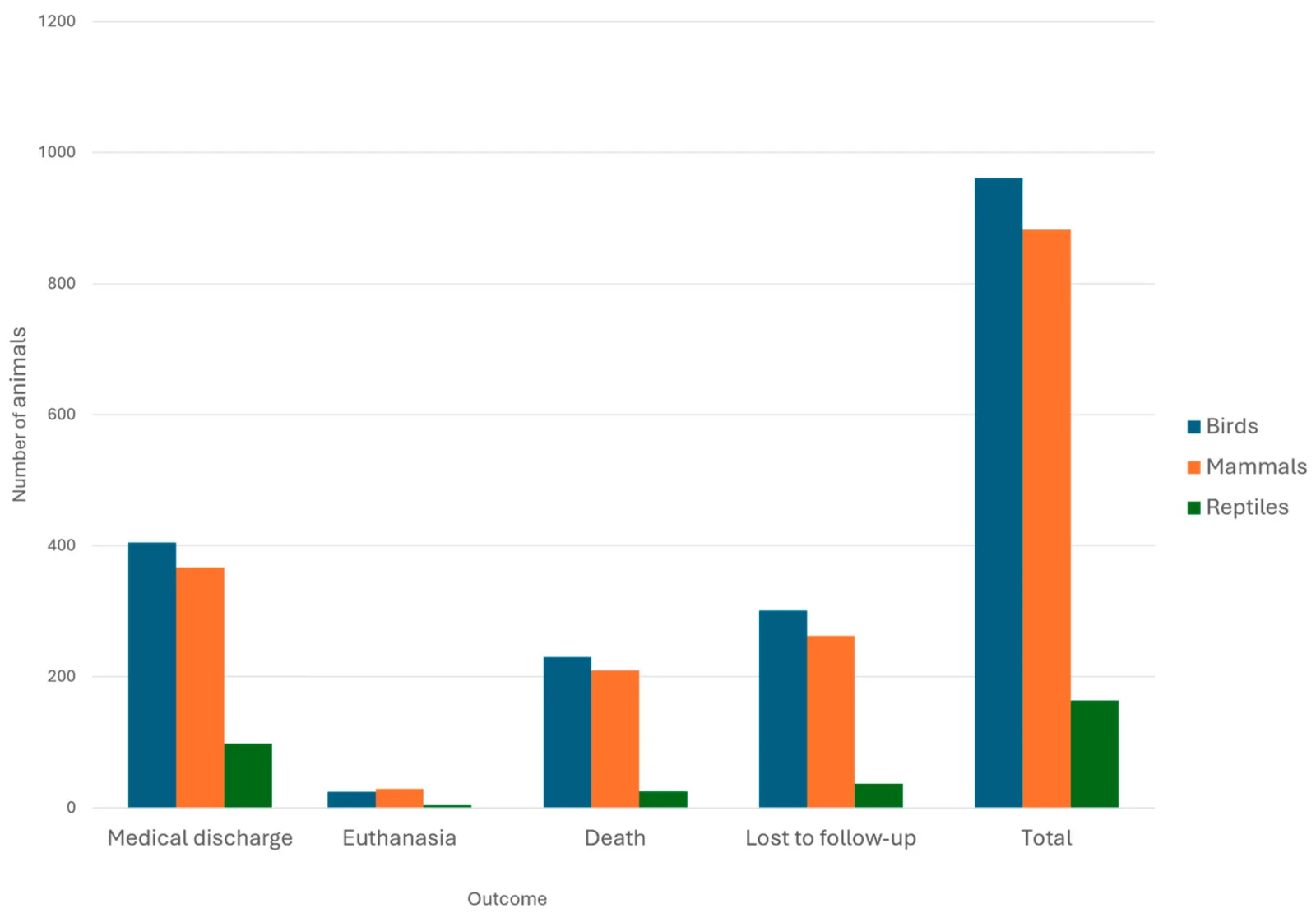
Outcomes of birds, mammals, and reptiles admitted to the referral center over an 11-year period.

Regarding outcomes, medical discharge was the most frequent final status in all classes, occurring in 405 birds, 367 mammals, and 98 reptiles. Death was also common, affecting 230 birds, 216 mammals, and 25 reptiles. Euthanasia was relatively infrequent, with 24 birds, 32 mammals, and 4 reptiles. Loss to follow-up was frequent among birds (302) and mammals (267), but less common in reptiles (37). Reptiles showed the highest apparent proportion of medical discharge and the lowest proportion of loss to follow-up, whereas birds and mammals had more variable outcomes across years. Figure 5 presents the outcomes by class across all years.

Over the study period, lost to follow-up declined significantly in birds (R^2^ = 0.824; *p* = 0.0001), mammals (R^2^ = 0.673; *p* = 0.002), and reptiles (R^2^ = 0.808; *p* = 0.0002). In birds, death (R^2^ = 0.461; *p* = 0.022) and euthanasia (R^2^ = 0.393; *p* = 0.039) also declined, whereas euthanasia increased in reptiles (R^2^ = 0.512; *p* = 0.013). No other outcome categories showed significant temporal trends (*p* > 0.05).

## 4. Discussion

The hypothesis that admission frequencies would differ among non-traditional or “exotic” pet classes, with birds being the most frequently admitted class in all years, was only partially supported. Birds had significantly higher admissions than the other classes from 2014 to 2019; however, from 2020 to 2024, admissions of birds and mammals did not differ significantly. The marked reduction in admissions in 2020, followed by partial recovery, occurred across all three vertebrate classes. Although the study did not investigate its causes, this pattern coincided temporally with the COVID-19 pandemic, when mobility restrictions, reduced veterinary service availability, and financial constraints may have limited access to specialized care [17]. As these factors were not evaluated, this interpretation remains speculative.

Considering all animals recorded in the present study, birds (47.9%) and mammals (44.0%) accounted for the largest proportions, whereas reptiles represented a smaller proportion (8.2%), and no amphibians were recorded. The distribution of the most commonly represented “exotic” pets has varied across studies. In the United Kingdom, data from UK Pet Food indicated that birds were the fourth most commonly kept type of pet, present in approximately 2.7% of households [8]. Similarly, a survey of small animal practitioners in Istanbul, Turkey, found that birds were the most common “exotic” pets, followed by turtles, other reptiles, and fish [6]. A study from Mexico also identified birds as the most commonly kept “exotic” pets, followed by reptiles and small mammals [18]. However, other studies from the same university in Mexico have reported different distributions. Between 2005 and 2010, reptiles and mammals were the most frequently presented groups, whereas a more recent study covering 2009–2019 reported a predominance of mammals, followed by reptiles, with birds and other groups accounting for smaller proportions of presentations [9,10]. These differences may reflect variation in geographic location, study period, population characteristics, and the type of data collected. In Ireland, for example, a survey found that reptiles and birds were among the most commonly owned “exotic” pets reported by veterinary professionals [19]. Despite the relatively low representation of reptiles in the present study, this finding should not be interpreted as indicating limited conservation concern. In Brazil, challenges in regulating both the legal and illegal trade of native and exotic reptiles may increase the risks associated with the legalization and expansion of the reptile pet trade, including biological invasions, overexploitation of native species, illegal trade, and species extinction [20].

Within each vertebrate class, the most commonly admitted species in our study were cockatiels, rabbits, and red-footed tortoises among birds, mammals, and reptiles, respectively. This pattern partially aligns with findings from a Guatemalan study, in which rabbits, chelonians (*Trachemys* sp. and *Kinosternon* sp.), budgerigars (*Melopsittacus undulatus*), guinea pigs, and hamsters were the most frequently treated “exotic” animals in veterinary clinics [21]. Notably, rabbits and chelonians were common in both studies, while differences were observed in the predominant bird and small mammal species. In contrast, except for rabbits, the findings of a study conducted at a teaching hospital in Mexico differed from those of the present study, with red-eared sliders (*Trachemys scripta elegans*) and yellow-cheeked parrots (*Amazona autumnalis*) accounting for the highest proportions of admissions among reptiles and birds, respectively [10].

### 4.1. Birds

The cockatiel accounted for 47.76% of cases in the present study, followed by the domestic chicken, the second most frequently admitted species, at 15.19%, and the blue-fronted Amazon at 8.01%. A study identified the most common species of “pet” birds by order of prevalence, with the cockatiel being the most prevalent in Eastern Australia and the western United States; the budgerigar (*Melopsittacus undulatus*) in Western Canada; the red-lored Amazon (*Amazona autumnalis*) in Central America and Mexico; the orange-winged Amazon (*Amazona amazonica*) in Peru; and the grey parrot (*Psittacus erithacus*) in the Netherlands and South Africa [18]. The cockatiel, native to Australia, has an average lifespan of 15–20 years in captivity [22], although considerably longer lifespans of 28 and 35 years have been documented [23]. The blue-fronted Amazon parrot, widely distributed in Brazil, may live up to approximately 60 years in captivity [24], whereas the domestic chicken typically has a shorter lifespan of 5–10 years [25]. Although these species differ markedly in longevity, the present study did not evaluate age as a factor associated with hospital presentation frequency. Therefore, no inference can be made regarding whether lifespan influenced the higher representation of cockatiels, blue-fronted Amazons, or chickens in this caseload.

Most birds presented conditions related to the digestive system (*n* = 383), integumentary system (*n* = 310), and musculoskeletal system (*n* = 213), a pattern that appears consistent with conditions commonly reported in psittacine species. In cockatiels, a significant proportion of health problems are closely associated with poor diet and inadequate husbandry, in addition to infectious diseases such as polyomavirus, psittacosis, candidiasis, and Pacheco’s disease which can be fatal [22]. In addition, a retrospective study conducted at the University of León Hospital (2018–2022) found that birds were the most commonly admitted ‘exotic” pets, with most visits related to emergencies and unspecified musculoskeletal disorders; notably, one-third of the birds were euthanized [26]. Therefore, the pattern of the main affected body systems and sense organs identified in our study was likely influenced by species-related predispositions, diet, and husbandry practices.

In addition, several body system/sense organ categories in birds, including respiratory, sense organ–eye, musculoskeletal, digestive, and integumentary conditions, showed declining annual frequencies in the present study. However, these declines may not reflect reduced disease occurrence but rather the overall decrease in admissions after 2020. Because the analysis used annual case counts rather than rates adjusted for admissions, changes in caseload volume may have influenced these trends

### 4.2. Mammals

Among mammals, the four most frequently admitted were rabbits (52.38%), guinea pigs (17.57%), brown rats (10.77%) and Syrian hamsters (6.00%). Rabbits, followed by guinea pigs, are considered popular “exotic” pets in the United Kingdom, United States, and Mexico [10,15,27], which also corresponds to the current study. The small size of rabbits, along with their ability to socialize and the potential for training, are some of the reasons they are chosen as apartment pets [27].

In the present study, the most affected systems in mammals were the digestive system (*n* = 303), followed by the integumentary (*n* = 288), musculoskeletal (*n* = 126), and reproductive systems (*n* = 105). Rabbits are prone to various conditions, including dental and gastrointestinal diseases, with digestive disorders caused by infectious or non-infectious factors [15,28]. Skin lesions are also common, such as alopecia caused by mites, abscesses, or even myiasis [28,29]. Within a population of over 2000 pet rabbits receiving primary veterinary care in England, the most prevalent disorder groups were dermatological, oral, gastrointestinal/abdominal, ocular, and parasitic [30]. Guinea pigs may also develop dental disease, gastrointestinal hypomotility, gastric dilatation/volvulus, and skin disorders such as pododermatitis [15,31]. A study of 51,622 guinea pigs under UK veterinary care found that claw/nail, skin, and ophthalmic disorders were the most prevalent at the disorder-group level [32]. On the other hand, rats can present with a wide range of diseases, including mammary tumors, respiratory infections, congestive heart failure, chronic progressive nephropathy, skin diseases such as pododermatitis and ectoparasite infestations, acquired dental disease, and musculoskeletal disorders, among others [33]. In addition, the medical and surgical conditions most frequently described in hamsters include neoplasms, dental disorders, respiratory and renal diseases, abdominal cysts, heart disease, reproductive disorders, enteropathy with diarrhea, and impaction of the buccal pouches [34]. Of the 24 known hamster species, five are commonly kept as pet exotics, including the Syrian hamster, dwarf hamsters (*Phodopus sungorus*, *Phodopus campbelli*, and *Phodopus roborowskii*), and the Chinese hamster (*Cricetulus griseus*) [34]. In the present study, two species were identified, with a predominance of the Syrian hamster over the Chinese hamster. Therefore, the predominance of digestive, integumentary, musculoskeletal, and reproductive conditions among mammals likely reflects the combined contribution of the most commonly admitted species rather than a uniform disease pattern across all mammal species. In addition, only integumentary disorders showed a significant declining trend in mammals. As with birds, this finding should be interpreted in the context of changes in the total number of admissions over time. The absence of significant trends in the remaining categories does not necessarily indicate stable disease prevalence, as the study was not designed to estimate population-based incidence or prevalence.

### 4.3. Reptiles

Among reptiles, tortoises were the most frequently admitted, accounting for 70.12% of cases—113 red-footed tortoises and 2 yellow-footed tortoises (*Chelonoidis denticulata*). Additionally, 18.90% of cases involved turtles, including the black-bellied slider (*n* = 25), red-eared slider (*Trachemys scripta*, *n* = 4), and unidentified *Trachemys* species (*Trachemys* sp., *n* = 2). This pattern differed from a study conducted in Brazil, which found that snakes were the main group of reptiles kept as exotic pets [14]. This discrepancy may reflect methodological differences, as that study used online semi-structured questionnaires distributed to 719 pet reptile keepers in Facebook groups. Since identifying health changes in snakes is more challenging, this may explain the lower number of snake presentations in the current study, with only 11 admissions: eight corn snakes (4.87%) (*Pantherophis guttatus*), two boas (1.21%) (*Boa constrictor*), and one ball python (0.60%) (*Python regius*).

In the present study, the most commonly affected systems in reptiles were the integumentary (*n* = 57), musculoskeletal (*n* = 41), and digestive (*n* = 40) systems. Many chelonians present clinical issues related to inadequate husbandry and nutrition, parasitic infections, and trauma [35]. Of 2040 chelonians reviewed under UK primary veterinary care, 94.26% were tortoises, 3.63% terrapins and 2.11% turtles, with the most frequently recorded disorders being beak abnormalities, overgrown nails and shell abnormalities [36]. In our study, these abnormalities were categorized as part of the integumentary system. About the significant decline in integumentary records over time, interpretation should be cautious given the small number of reptile cases and potential annual variation in the regression results.

### 4.4. Outcomes

Medical discharge was the most frequent outcome across all vertebrate classes, indicating that many animals were successfully managed after treatment or hospitalization. Death and loss to follow-up were also common, particularly in birds and mammals. However, loss to follow-up declined significantly across all three classes, potentially reflecting improved owner communication, record-keeping, or adherence to follow-up recommendations, although these factors could not be assessed retrospectively. Death and euthanasia also declined in birds, while euthanasia increased in reptiles. Because reasons for outcomes were not evaluated, these trends should not be interpreted as changes in prognosis or quality of care.

### 4.5. Limitations

This study has several limitations that should be considered when interpreting the results. First, the data were collected from a tertiary referral center, and therefore the study population may not be representative of the species and cases encountered in first-opinion veterinary practice. Second, because only privately owned animals presented for veterinary care were included, species more commonly kept as pets, easier to transport, or more likely to receive specialized veterinary attention may be overrepresented. Third, potential risk factors such as age, sex, husbandry conditions, nutrition, or owner-related factors were not evaluated. Therefore, further studies are needed to evaluate these variables and better clarify their influence on the findings.

## 5. Conclusions

The study revealed that, at the Center for Medicine and Research in Wild Animals (CEMPAS), birds and mammals were the most frequently admitted non-traditional or “exotic” pets, whereas reptiles were the least represented class. Bird admissions exceeded those of the other classes during the first six years of the study, whereas from 2020 onward, bird admissions did not differ significantly from mammal admissions. Cockatiels, rabbits, and red-footed tortoises were the most commonly admitted species within their respective classes. Across the three vertebrate classes, the digestive and integumentary systems were the most frequently affected. Medical discharge was the predominant outcome, although death and loss to follow-up were also common. These findings provide region-specific information on the profile of non-traditional or “exotic” pet admissions and may help inform veterinary clinical practice and future research. A limitation of this study is that it focused on identifying affected body systems and sense organs rather than characterizing specific lesions. Further studies are therefore needed to investigate lesion types and clinical conditions in greater detail within each class.

## Figures and Tables

**Table 1:** Annual distribution of birds, mammals, and reptiles admitted to the referral center from 2014 to 2024, with 95% confidence intervals.

	Birds	Mammals	Reptiles	
Year	Number (%) [95% CI]	Number (%) [95% CI]	Number (%) [95% CI]	Total (Number)
2014	117 (52.47) [45.70–59.18]	90 (40.36) [33.86–47.10]	16 (7.17) [4.16–11.33]	223
2015	110 (48.25) [41.80–55.16]	104 (45.61) [39.21–52.54]	13 (5.73) [3.08–9.60]	227
2016	121 (52.61) [45.93–59.22]	91 (39.57) [33.18–46.23]	18 (7.83) [4.72–12.08]	230
2017	105 (51.47) [44.37–58.53]	83 (40.69) [33.88–47.81]	16 (7.84) [4.54–12.41]	204
2018	117 (51.32) [44.62–57.99]	88 (38.60) [32.27–45.23]	23 (10.09) [6.53–14.78]	228
2019	102 (50.75) [43.63–57.85]	90 (44.78) [37.79–51.97]	9 (4.48) [2.07–8.30]	201
2020	43 (42.16) [32.44–52.37]	48 (47.06) [37.10–57.18]	11 (10.78) [5.52–18.45]	102
2021	44 (35.20) [26.87–44.24]	73 (58.40) [49.24–67.13]	8 (6.40) [2.81–12.19]	125
2022	56 (47.06) [37.81–56.46]	52 (43.70) [34.64–53.06]	11 (9.24) [4.71–15.92]	119
2023	55 (38.19) [30.22–46.68]	74 (51.39) [42.93–59.80]	15 (10.42) [5.96–16.61]	144
2024	91 (44.61) [37.67–51.71]	89 (43.63) [36.72–50.77]	24 (11.76) [7.66–17.00]	204
Total	961 (47.88)	882 (43.95)	164 (8.17)	2007

**Table 2:** Bird species admitted to the referral center, categorized by year.

Species	2014	2015	2016	2017	2018	2019	2020	2021	2022	2023	2024	Total
*Agapornis* sp. (lovebird)	1	3	2	4	2	2	1		2	2	1	20
*Amazona aestiva* (blue-fronted Amazon)	10	12	7	11	12	6	3	2	5	2	7	77
*Amazona amazonica* (orange-winged Amazon)		1										1
*Anser anser* (greylag goose)	2	2	2	3	1	3	2		1	2	2	20
*Anas georgica* (yellow-billed pintail)									1			1
*Anas platyrhynchos* (mallard)	2		4	1		1	1					9
*Ara ararauna* (blue-and-yellow macaw)	3	2	2	1	3	4		1			1	17
*Aratinga jandaya* (jandaya parakeet)										1		1
*Brotogeris chiriri* (yellow-chevroned parakeet)	1				2					1		4
*Cacatua alba* (white cockatoo)						1						1
*Cairina moschata* (muscovy duck)											3	3
*Cariama cristata* (red-legged seriema)									1			1
*Carduelis cucullata* (red siskin)			1									1
*Chaetura meridionalis* (sick’s swift)									4			4
*Chrysolophus pictus* (golden pheasant)											1	1
*Columba livia* (rock pigeon)				1	1			1			1	4
*Coragyps atratus* (black vulture)	1											1
*Coturnix coturnix* (common quail)				2	2	2	1	1				8
*Cyanoloxia brissonii* (ultramarine grosbeak)	1			2	4	1						8
*Dromaius novaehollandiae* (emu)											1	1
*Eclectus roratus* (ecletus parrot)			1									1
*Gallus gallus domesticus* (chicken)	6	11	25	14	17	21	10	10	7	6	19	146
*Gnorimopsar chopi* (chopi blackbird)			1	1								2
*Icterus jamacaii* (campo troupial)					1							1
*Meleagris gallopavo* (wild turkey)	1	2		3	2						2	10
*Melopsittacus undulatus* (budgerigar)	2	3	1	1	3	3	4	2	3	3	1	26
*Neopsephotus bourkii* (bourke’s parrot)										1		1
*Numida meleagris* (helmeted guineafowl)		2	1			2	1		1			7
*Nymphicus hollandicus* (cockatiel)	69	46	56	52	55	52	18	22	22	27	40	459
*Oryzoborus angolensis* (lesser seed finch)		2	3		1							6
*Passer domesticus* (house sparrow)					1							1
*Pavo spp.* (peafowl)		5	1	2	1	1						10
*Pavo cristatus (Indian peafowl)*								3		2		5
*Pitangus sulphuratus* (great kiskadee)	2											2
*Platycercus eximius* (eastern rosella)									2			2
*Psittacara leucophthalmus* (white-eyed parakeet)	5	10	7	6			1		1	1	3	34
*Psittacus erithacus* (grey parrot)		1										1
*Psittacula krameria* (rose-ringed parakeet)		3	3		2	2					3	13
*Ramphastos toco* (toco toucan)	3	1	1	1					2			8
*Saltator similis* (green-winged saltator)	1	1			2							4
*Serinus canaria* (Atlantic canary)	6	2			2		1	1	4	3	4	23
*Sicalis flaveola* (saffron finch)		1	1		1			1		1		5
*Spatula querquedula* (garganey)											1	1
*Sporophila caerulescens* (double-collared seedeater)	1		1							1		3
*Struthio camelus* (common ostrich)			1			1						2
*Tyrannus melancholicus* (tropical kingbird)										1		1
*Tyrannus savana* (fork-tailed flycatcher)					1							1
*Zenaida auriculata* (eared dove)					1					1	1	3

**Table 3:** Mammal species admitted to the referral center, categorized by year.

Species	2014	2015	2016	2017	2018	2019	2020	2021	2022	2023	2024	Total
*Alouatta* spp. (howler monkey)			1									1
*Atelerix albiventris* (African pygmy hedgehog)	1					1	1					3
*Callithrix jacchus* (common marmoset)	1	1										2
*Callithrix penicillata* (black-tufted marmoset)		2			2	2		1				7
*Callithrix* sp. (marmoset)	2	6	3			1		2				14
*Cavia porcellus* (guinea pig)	13	16	17	14	17	14	13	15	4	15	17	155
*Coendou spinosus* (Paraguayan hairy dwarf porcupine)									1			1
*Chinchilla lanigera* (long-tailed chinchilla)	3	2	7	3	1					1		17
*Cricetulus griseus* (Chinese hamster)	2			5	12	1	5	5	1			31
*Didelphis albiventris* (white-eared opossum)	1									2	2	5
*Lama glama* (llama)								1			2	3
*Meriones unguiculatus* (Mongolian gerbil)			1	2			1	1	1			6
*Mesocricetus auratus* (Syrian hamster)	21	5	11			11			1	3	1	53
*Mustela putorius furo* (ferret)		1		1	2							4
*Mus musculus* (house mouse)	1					1					2	4
*Nasua nasua* (South American coati)						1				1		2
*Oryctolagus cuniculus* (European rabbit)	40	62	42	49	49	43	20	32	36	41	48	462
*Phodopus campbelli* (Campbell’s dwarf hamster)									1	2	5	8
*Rattus norvegicus* (brown rat)	5	6	9	9	5	14	7	14	7	9	10	95
*Sapajus* spp. (capuchin monkey)						1	1	2			1	5
*Sciurus aestuans* (Brazilian squirrel)		1										1
*Sus scrofa* f. *domestica* (domestic pig)		2										2
*Vicugna pacos* (alpaca)											1	1

**Table 4:** Reptile species admitted at the referral center, categorized by year.

Species	2014	2015	2016	2017	2018	2019	2020	2021	2022	2023	2024	Total
*Boa constrictor* (boa constrictor)										2		2
*Chelonoidis carbonaria* (red-footed tortoise)	12	8	11	14	13	7	9	4	10	9	16	113
*Chelonoidis denticulata* (yellow-footed tortoise)											2	2
*Iguana iguana* (green iguana)	2											2
*Pantherophis guttatus* (corn snake)			1	1	1			2		2	1	8
*Pogona vitticeps* (central bearded dragon)	1											1
*Polychrus acutirostris* (spiny monkey lizard)			1									1
*Python regius* (ball python)										1		1
*Trachemys dorbigni* (black-bellied slider)	1	2	5	1	9	2	2			1	2	25
*Trachemys scripta* (pond slider)								1	1		2	4
*Trachemys* sp. (slider turtle)								1			1	2
*Salvator merianae* (Argentine black and white tegu)		1										1
*Furcifer pardalis* (panther chameleon)		2										2

## Data Availability

The data supporting the findings of this study are available within the article.
